# P-775. Trends in Urosepsis-Related Mortality Among Adults ≥ 25 Years of Age in the United States from 1999 to 2020: Insights from Retrospective CDC-Wonder Analysis

**DOI:** 10.1093/ofid/ofaf695.986

**Published:** 2026-01-11

**Authors:** Kenneth Hannan, Hamza Asif, Saadia Ashraf

**Affiliations:** University of Louisville Hospital, Louisville, Kentucky; University of Louisville Hospital, Louisville, Kentucky; Khyber Teaching Hospital, Peshawar, Pakistan, Peshawar, North-West Frontier, Pakistan

## Abstract

**Background:**

Urosepsis is a significant cause of morbidity and mortality. Despite its impact, long-term trends in urosepsis-related mortality in the United States (U.S.) have not been thoroughly examined. This study analyzes temporal trends and geographical variations in urosepsis-related mortality in adults ≥ 25 years of age from 1999 to 2020.

**Figure:**
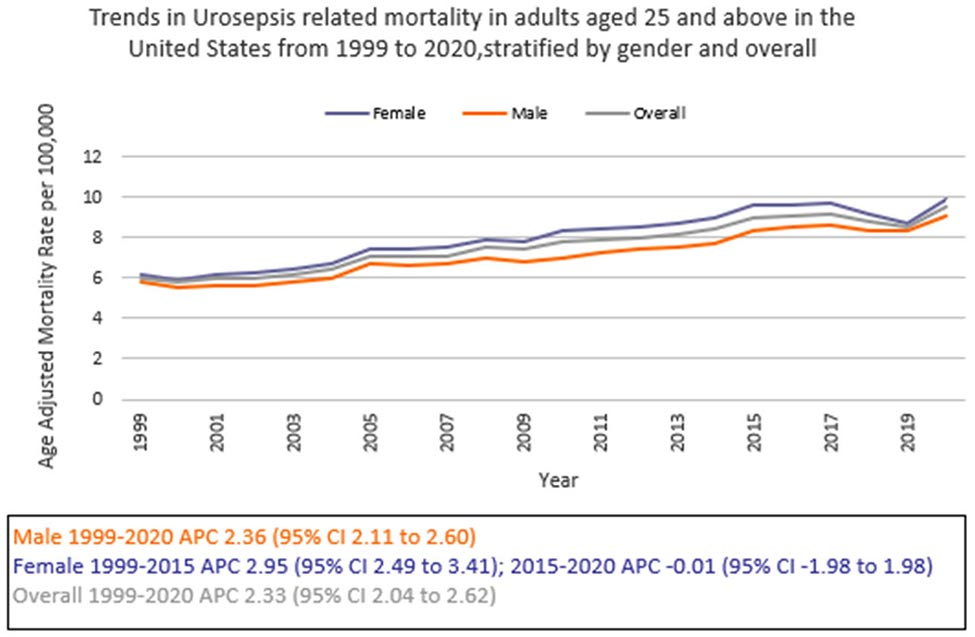

**Figure:**
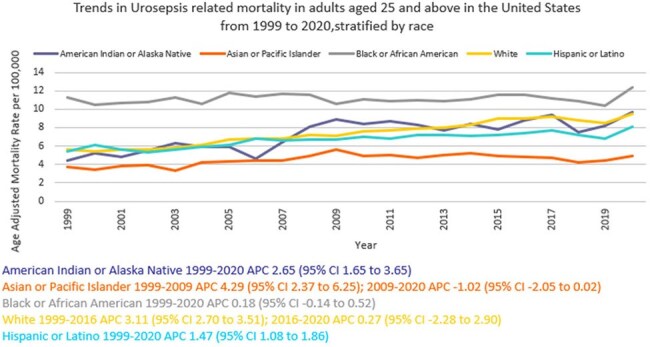

**Methods:**

We analyzed death certificate data from the CDC WONDER (Centers for Disease Control and Prevention Wide-Ranging Online Data for Epidemiologic Research) database between 1999 and 2020. Urinary tract infection (UTI) with septicemia-related deaths in adults ≥ 25 years of age were examined, using the 2000 U.S. standard population for age standardization. Mortality rates were expressed as age-adjusted mortality rates (AAMR) per 100,000 population. Joinpoint regression was used to assess trends and calculate annual percentage change (APC), stratified by year, sex, race/ethnicity, census region, geographical location and states.

**Figure:**
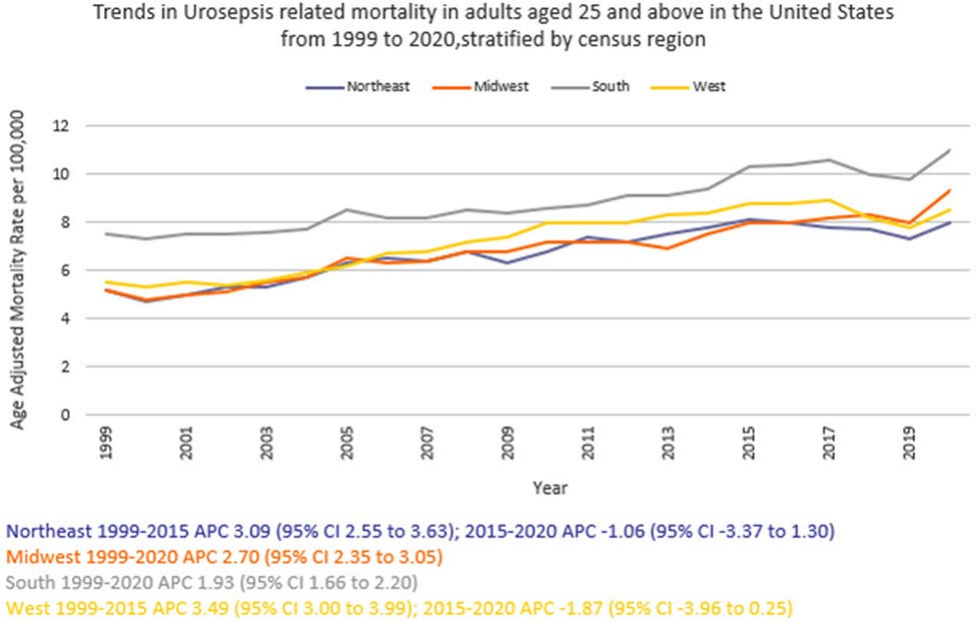

**Figure:**
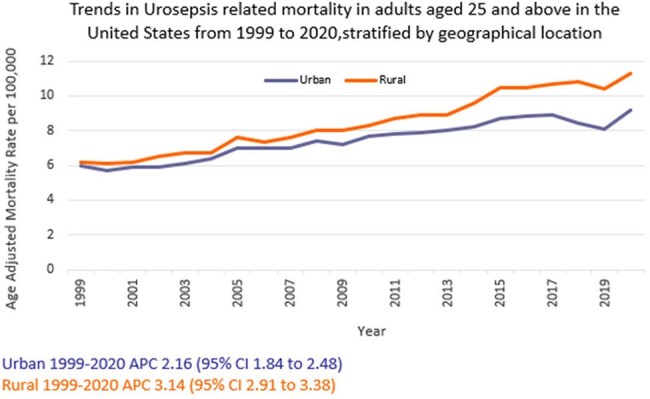

**Results:**

A total of 368,313 urosepsis-related deaths occurred between 1999 and 2020. The AAMR increased from 6.0 in 1999 to 9.5 in 2020 (APC 2.33; 95% CI: 2.04 to 2.62). Women consistently had higher AAMRs than men (8.1 vs.7.3). Non-Hispanic (NH) Blacks or African Americans had the highest AAMR (11.2) followed by NH American Indians or Alaska Natives (7.6), NH Whites (7.5), Hispanics or Latinos (6.9), and NH Asians or Pacific Islanders (4.6). Regional variations in AAMR were also significant, with the highest rates in the South (9) followed by West (7.4), Midwest (6.9), and Northeast (6.8). Residents of rural areas consistently had higher AAMRs than those of urban areas (8.6 vs 7.6). Geographically, AAMRs ranged from 13.3 in Tennessee to 3.5 in Hawaii.

**Conclusion:**

From 1999 to 2020, urosepsis-related mortality in the U.S. increased overall, with persistent disparities noted among women, NH Blacks or African Americans, and residents of the South and rural areas. These trends highlight the need for enhanced public health surveillance to better understand the scope of Urosepsis-related mortality and to identify high-risk demographic and regional subgroups for targeted interventions.

**Disclosures:**

All Authors: No reported disclosures

